# Metastatic and triple-negative breast cancer: challenges and treatment options

**DOI:** 10.1007/s13346-018-0551-3

**Published:** 2018-07-05

**Authors:** Sumayah Al-Mahmood, Justin Sapiezynski, Olga B. Garbuzenko, Tamara Minko

**Affiliations:** 1 Department of Pharmaceutics, Ernest Mario School of Pharmacy, Rutgers, The State University of New Jersey, 160 Frelinghuysen Road, Piscataway, NJ 08854-8020 USA; 2 Rutgers Cancer Institute, New Brunswick, NJ 08903 USA; 3 Environmental and Occupational Health Sciences Institute, Rutgers, Rutgers, The State University of New Jersey, Piscataway, NJ 08854 USA

**Keywords:** Liposomes, EGFR, siRNA, Gefitinib, Combinatorial treatment of breast cancer

## Abstract

The major current conventional types of metastatic breast cancer (MBC) treatments include surgery, radiation, hormonal therapy, chemotherapy, or immunotherapy. Introducing biological drugs, targeted treatment and gene therapy can potentially reduce the mortality and improve the quality of life in patients with MBC. However, combination of several types of treatment is usually recommended. Triple negative breast cancer (TNBC) accounts for 10–20% of all cases of breast carcinoma and is characterized by the low expression of progesterone receptor (PR), estrogen receptor (ER), and human epidermal growth factor receptor 2 (HER2). Consequently, convenient treatments used for MBC that target these receptors are not effective for TNBC which therefore requires special treatment approaches. This review discusses the occurrence of MBC, the prognosis and predictive biomarkers of MBC, and focuses on the novel advanced tactics for treatment of MBC and TNBC. Nanotechnology-based combinatorial approach for the suppression of EGFR by siRNA and gifitinib is described.

## Introduction

Breast cancer is a heterogeneous and a complex disease [1–5]. It is composed of different biological subtypes, which are human epithelial growth receptor type 2 (HER-2), luminal A, luminal B, claudin-low, and basal-like. These five subtypes have different abilities to metastasize to distant organs, specific pathways with the preferred metastatic sites, and different survival response after relapse [6]. Patients who have the luminal subtypes of breast cancer frequently for example have bone relapses; however, breast cancer of basal subtype often metastasizes to the lungs and brain, and cannot reach statistical significance in patients with liver relapse [2, 4]. The biological subtypes of breast tumor can be defined by immunohistochemical (IHC) biomarkers or gene expression profiles [2, 7]. In general, the standard prognostic and predictive factors for breast cancer disease are human epidermal growth factor receptor 2 (HER2), progesterone receptor (PR), estrogen receptor (ER), and proliferation (Ki-67) status [4, 8]. The choice of local or systemic treatment can vary related to these different subtypes of breast cancer [7]. Breast cancer can spread to other sites of the body resulting in metastatic breast cancer (MBC) [3]. Between 6 and 60% of patients with breast cancer were diagnosed early with MBC [1, 2, 6, 9–11]. MBC is the second leading cause of death among women in the USA [12]. Age, race, ethnicity, endogenous hormones, menopause, histological status of cells, smoking, first degree relative, number of metastatic sites, duration of breast feeding, mutation, and the underlying biology of the tumor such as grade and size of the primary tumor can increase the chance of MBC occurrence [13–23]. The main sites of breast cancer to spread are lungs, bones, liver, brain, soft tissue, and adrenal glands [4, 11, 24, 25]. This manuscript reviews (a) process of metastatic breast cancer occurrence, (b) the prognostic factors that detect or imply the occurrence of MBC, (c) the possible models or theories of the occurrence of MBC, and finally (d) the treatment of MBC. It also describes a novel approach for treatment of triple-negative breast cancer.

## Metastatic breast cancer

**Fig. 1:**
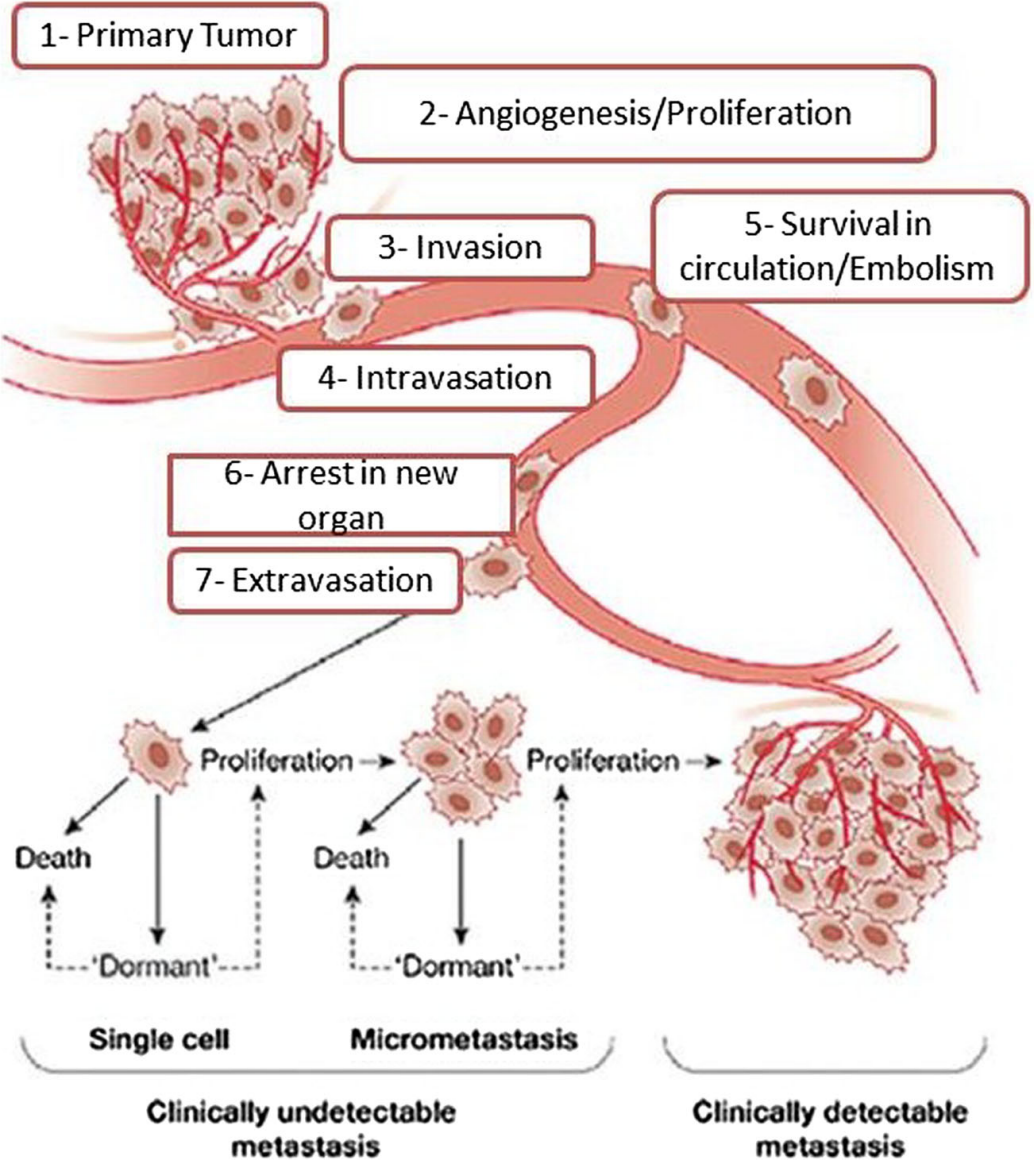
Major steps of breast cancer metastasis formation. Modified with permission from [39]

MBC process is a complex multistep process that includes many steps of dynamic interactions between cells of the tumor and the host resulting in leaving of tumor cells from their primary site and metastasis to a distant area. Figure 1 shows the different physiological activities of MBC from the primary tumor to the secondary site [26–29]. It should be stressed that similar mechanisms of metastasis are involved in the spreading of primary cancer cells via lymphatic system, although the involvement of lymphangiogenesis in this process is controversial [30]. Metastasis process is also known as non-passive or nonlinear process because it is like loops between cells of the tumor and cells of the host in the tumor microenvironment. When the tumor is formed, it grew and proliferated overcoming the cellular restrictions that leads to disrupt the local homeostasis and affected hypoxia, acidosis, as well as systemic and tissue pressures. During the initial phases of tumor proliferation, the host activates tissue repair mechanisms by providing the neoplasm with a supply of nutrients vascularization, removing of waste, and escaping route for the prospective metastatic cell in an attempt to compensate changes in the primary site. At the same time, the physical stress of the growing lesion initiates an inflammatory response that mobilizes bone marrow-derived cells (BMDCs) and other leukocytes to the primary and potential secondary sites. This uncommon and unnatural mixture of cells results in a reactive microenvironment as well as a suitable environment of cytokines, growth factors, and extracellular matrix (ECM) proteins. The re-modeling of ECM proteins within the interstitial space is a marker of highly invasive tumors. In the case of tumor, the inflammation fails to resolve and stimulate the occurring involvement of the immune-regulatory cells leading to decrease in the response of antitumor immune system [26, 31–33]. Later, these tumor cells acquire more mutant alleles that enable them to spread and seed new colonies at different anatomical sites that are distant from the primary tumor mass. Activation of oxidoreductase enzymes and latent proteases alter topology of ECM and improve the invasion of tumor cell by the exposure of cryptic adhesive sites and the release of pro-migratory peptides. Therefore, the host cells can develop genetic changes that enable them to carry these mutant alleles to offspring of people within the primary tumor mass. Ligation adhesion receptors of tumor cell to this modified ECM simulating intracellular pathways that induce invasion through the stroma and finally into the lymphatics or bloodstream [31, 34, 35].

It was also reported that MBC may occur through the lymphatic system [30]. The spread of cancer cells by lymphatic vessels to lymph nodes sites is an important prediction of tumor aggressiveness for most human tumors. On the other hand, the tumor cell must resist the physical stress caused by loss of vascular turbulence and adhesion before its arrest in a distant capillary bed in circulation. During transit, tumor cells can form a bolus with platelets, which protects them from the stresses of shear flow and enhances their sensitivity to chemokine gradients. Among combination of physical obstruction, attractive chemokine gradients, and the complementary adhesive contacts, the cancer bolus is attracted and became surrounded by capillaries of the secondary site. As a result, lodged cancer cells may grow as an intravascular metastasis or may extravasate into the secondary tissue [26, 31, 36].

In the secondary sites, cancer cells are arranged in small capillaries and deformed to fit the vasculature in the new sites according to the blood pressure in the new organ and the size restrictions. Cancer cells can occur in the secondary sites as small pre-angiogenic metastases, solitary cells, or large vascularized metastases. Only a subset of these cells can persist and the remainder of cells (micrometastases) might either go into a state of dormancy (dormant solitary cells are cells that are undergoing neither apoptosis nor proliferation) or die during every step of the metastatic process. In general, micrometastases and solitary cells are clinically undetectable and only a proportion of vascularized metastases are clinically detectable [26, 34].

### Triple-negative breast cancer

Several pathways are involved in the development of triple-negative breast cancer (TNBC) from basal-like breast cancer cells. The main of them include the loss in the expression of several receptors by BRCA1-related pathway or random mutation(s) [37]. TNBC accounts for 10–20% of all cases of breast carcinoma and is characterized by the low expression of progesterone receptor (PR), estrogen receptor (ER), and HER2. The development of metastases in TBNC represents a highly complex and poorly understood process that includes multiple steps such as genetic and epigenetic alterations, angiogenesis, tumor–stroma interactions, intravasation through the basement membrane, survival in the circulation, and extravasation into distal tissues [38]. Patients with TNBC have a relatively poor outcome and cannot be treated with endocrine therapy or therapies targeted to HER2. Consequently, this type of metastatic breast cancer requires special treatment approaches. In addition, the overexpression of EGFR protein specific to TNBC (when compared with other subtypes of breast cancers) usually increase resistance of this type of cancer cells to conventional therapies [39]. Therefore, the suppression of this protein potentially can enhance the efficacy of treatment of TNBC. Small interfering RNA (siRNA) targeted to EGFR mRNA can be used for this aim. However, it is known that naked siRNA is not stable in the blood stream and inside cancer cells. Moreover, it possesses a very poor ability to penetrate inside cancer cells. Fortunately, nanotechnology approaches can be used for effective delivery of siRNA as well as conventional anticancer therapies inside TNBCs. Such approach proposed in our laboratory is described below (please see section 5.6.1.4).

## Prognostic and predictive factors of MBC

Most deaths of women with breast cancer arise due to the metastatic behavior of breast cancer and not as a result of the primary tumor growth. Consequently, prognostic factors can be successfully used to identify patients at high risk of metastatic breast cancer and to select a most effective treatment individually for each cancer patient. Prognostic factors can be derived from the specific environment of the host and from the tumor itself [5]. These prognostic factors can be pathological factors such as histological grade of the tumor, size of the primary tumor, and deposit of the tumor in the draining lymph nodes of the primary breast cancer. Specific genes and corresponding proteins related to the development of breast cancer have been discovered recently. These genes/proteins are involved, *inter alia*, in controlling cell proliferation (such as c-erbB-2 and c-erbB-3), cell death (such as p53), cell differentiation (such as pS2, ERα, and PgR), and cell invasion (such as cathepsin D) in tissue-cultured systems. However, these molecular markers have more limited use than the pathological factors in predicting death of patient from metastatic disease because they can relate more to the growth of the primary tumor and not necessarily to the development of distant metastases [40, 41]. A retrospective study showed that patients younger than 35 years old with early stage of breast cancer following both mastectomy and breast-conserving surgery had a worse prognosis with higher risk for developing MBC and greater overall recurrence comparing to older patients. In addition, prediction of the age at diagnosis showed that patients who are older than 40 years can be more prone to have triple-negative breast cancer [42–44]. The rate of death due to breast cancer remains higher among African Americans than Caucasian in the USA and this may be associated with the nature of tumors. In addition, African American women patients more likely have hormone receptor-negative tumors, positive axillary nodes, and positive axillary nodes associated with smaller tumors comparing with Caucasian women patients. Moreover, African American women who are receiving neo-adjuvant chemotherapy showed worse progression-free survival than Caucasian women but the overall survival in these groups was similar [45–47]. Table 1 shows the main prognosis and predictive factors of MBC which will be briefly discussed below.

**Table 1:** Prognosis and predictive factors of MBC

No.	Prognostic and predictive factors
1	Axillary lymph nodal involvement
2	Tumor size
3	Estrogen receptor (ER) and progesterone receptor (PR) status
4	Circulating tumor cells (CTCs)
5	Lymphatic and vascular invasion (LVI)
6	Age at diagnosis
7	Race and ethnicity
8	Cathepsin D
9	Angiogenesis markers
10	Bone marrow micometastasis
11	Overexpression of the c-erb B-2 (HER2/neu) Proto-oncogen
12	Urokinase-type plasminogen activator (uPA) and plasminoge activator inhibitor type 1 (PAI-1)
13	Mutations of p53
14	Expression of topisomerase II-alpha (topo Iiα)
15	Proliferation markers
16	Gene expression profiling

### Axillary lymph nodal involvement and tumor size

Axillary lymph nodal involvement is an important factor to recognize the staging, prognosis, and treatment of progression-free survival (PFS) and overall survival (OS) of breast cancer. The common methods for determine the lymph node involvement in breast cancer are sentinel node biopsy (SLNB), clinical assessment, axillary dissection, and evaluation of imaging methods. The predictor of axillary lymph node metastasis in general should be easy reproducible, cost-effective, high accurate, and induces minimum side effects on patients. If lymph-node metastasis is present, there is high risk of metastasis while if there is no lymph-node involvement, a patient has a low risk of metastasis. In addition, the presence of more than four lymph-node metastasis is associated with very high risk of metastasis and generally predicts a poor prognosis [5, 11, 48, 49].

Size of the tumor plays an independent role in the prognosis of MBC especially in several cases like axillary lymph node and HER-2 statues. The large size of tumor generally means worse prognosis and higher risk of MBC than small size of tumor. The size of breast cancer ≤ 2 cm in patients younger than 40 years old generally indicates a relatively low risk of metastasis correlated with the presence of negative estrogen receptor status and axillary lymph node status. However, tumors with the size within 2–5 cm have high risk of metastasis while tumors a size more than 5 cm have very high risk of metastasis. About 80% of patients with tumors measuring ≤ 1 cm have better 20-year recurrence PSF when compared with 72% of patients with tumor size 1.1–2 cm [11, 42, 50, 51].

### Estrogen and progesterone receptor

Estrogen receptor (ER) and progesterone receptor (PR) are considered the most important prognosis factors even before the invention of hormonal therapy. ER-positive patients with node-negative breast cancer who were treated with local therapy showed higher PFS and OS within 5 years. Hormone receptor is strongly associated with hormonal/endocrine treatment; however, hormonal therapy is not useful in hormone receptor negative tumor cases. Moreover, the loss of either PR or ER in recurrent breast cancer will be related with poor response to hormonal/endocrine therapy [5, 41, 42, 52]. Table 2 shows the percentage distribution of estrogen and progesterone receptors.

**Table 2:** The distribution of estrogen and progesterone receptors in different groups of patients. Modified from [5]

Hormone receptor status (*n* = 155,890)	ER+/PR+	ER+/PR−	ER−/PR+	ER−/PR−
	64%	13%	3%	20%

### Circulating tumor cells and lymphovascular invasion

**Fig. 2:**
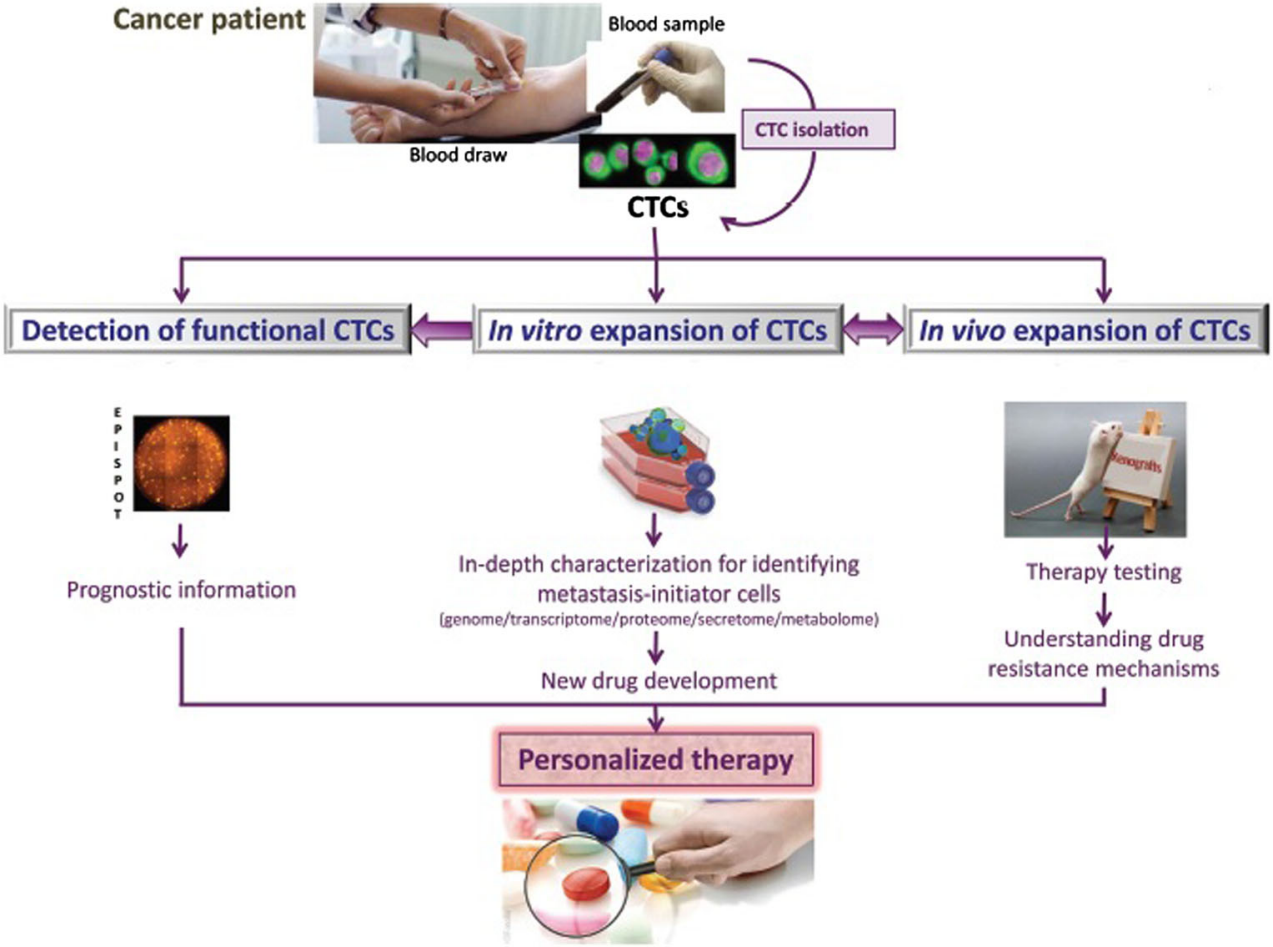
Role of CTCs in breast cancer in vitro and vivo. Modified and reproduced with permission from [55]

Circulating tumor cells (CTCs) are rare malignant cells that resulted or originated from the primary site. These cells circulate in the peripheral blood and can work as independent predictive and prognosis factor of early and advanced stage of breast cancer. The presence of more than five CTCs/7.5 ml of blood in MBC patients or more than one CTCs/7.5 ml of blood in non-metastasis patients can be predictive of poor PFS and OSC. As a result, CTCs can give information about the efficacy of the treatment by drawing a blood sample from cancer patient multiple times during his/her illness [53–62]. Figure 2 shows how CTCs works as prognosis factor for metastasis cells, treatment, and understanding drug resistance in breast cancer.

Lymphovascular invasion (LVI) involves both the lymphatic and blood vessel invasion lying within an endothelial-lined space in the area that surrounding the invasive tumor. LVI can be used as predictive factor for breast cancer patients. In addition, it is prognosis factor for lymph node, lymph node positive, and triple-negative breast cancer. About 23% of patients with early stage breast cancer showed vascular invasion [5, 42, 63–68].

### Cathepsin D, angiogenesis markers, and bone marrow micometastasis

According to their active site amino acid, the cathepsin family of lysosomal hydrolases can be divided into three sub-groups: cysteine (B, C, H, F, K, L, O, S, V, W, and X/Z), aspartate (D and E), and serine (G) cathepsins. Cathepsin D can be used as predictive factor for breast cancer. When the cathepsin D protein level exceeds 70 pmol/mg in patients with node-negative tumor, it is associated with poor prognosis [5, 69, 70].

The occurrence of tumor emboli in more than three blood vessels is most probably is associated by metastases. Microvessel density (MVD) is a common standard method of measuring angiogenesis of cancer. The high score of MVD in tumors in most cases indicates an easy and aggressive metastasis of cancer, and also is associated with a poor prognosis [11, 71–73].

Bone marrow micrometastasis refers to a small metastasis of less than 0.2 cm in diameter and can also include the tumor cells found in the bone marrow. The tumor cells usually can be found in 31% of lymph node-negative patients and 55% of lymph node-positive patients. The metastasis cells in the bone marrow are generally associated with poor clinical outcome in patients with breast cancer [5, 11, 74].

### Urokinase plasminogen activator and plasminogen activator inhibitor type 1

The urokinase-type plasminogen activator (uPA) system includes the serine protease uPA, its cell surface receptor urokinase-type plasminogen receptor (uPAR), and its serine inhibitors: plasminogen activator inhibitor type 1 (PAI-1) and plasminogen activator inhibitor type 2 (PAI-2). uPA is an extracellular matrix-degrading protease and PAI-1 representing the inhibitor of uPA is originally known as a blood-derived endogenous fast-acting inhibitor of uPA. Both uPA and PAI-1 can be used as independent prognostic factors for breast cancer patients since uPA has a role in the progression and metastasis of the tumor. In addition, uPA and PAI-1 are also included in cell signaling and can affect migration, chemotaxis, adherence, cell growth, anoikis, and survival. Moreover, uPA and/or PAI-1 can play a role in the physiological processes like blood clotting, wound healing, fibrinolysis, pregnancy, and tissue remodeling. Paradoxically, high protein levels of both these markers were related to high metastasis risk and poor PSF. In addition, uPA and PAI-1 are considered the best prognostic biomarkers for lymph node-negative breast cancer [5, 11, 75, 76].

### Mutation of p53

Tumor protein p53 is a tumor suppressor and plays an important role in the pathways of cellular stress response and regulation of the transcriptional programs which is important for suppressing the formation and progression of the tumor. The most common mutation of this gene involves the substitution of an arginine for a proline at codon position 72. The high rate of mutant p53 is related with cancer metastasis, tumor proliferation, and early death in node-negative breast cancer. Tumors with mutant p53 were also related with high local failure rate and poor response to systematic treatment such as tamoxifen [5, 77–79].

### Proliferation markers

It was shown that the S-phase fraction (SPF) value can predict the proliferation of the tumor to metastasis. The high level of SPF is associated with larger tumor size, worse tumor grades, and adversely with progression-free survival (PFS) and overall survival (OS) [5, 80, 81].

The high level of thymidine labeling index (TLI) is inversely correlated with the prognosis of node-negative tumors patients. In addition, low level of TLI in patients with early stage node-positive breast cancer is associated with better survival. Moreover, when the value of mitotic activity index (MAI) greater than 10 in patients with lymph node-negative breast cancer, it is correlate with greater rate of recurrence and mortality [5, 82, 83]. Antigen KI-67 is a nuclear protein that is associated with and may be necessary for cellular proliferation. It might be used as independent factor to measure the rate of proliferation. The high level of Ki-67 is associated with overexpression of HER2/neu, more lymph node involvement, and larger tumor size in patients with breast cancer. In addition, higher proliferating cell nuclear antigen (PCNA) was correlated with shorter relapse free and OS [84–87].

### Gene expression profiling

Because of the variation in the predictive markers of patient’s outcome that is determined by IHC, the analysis of gene patterns can be considered as an alternative method to define the treatment efficacy and its outcome. Assessment of gene array can be assessed by a DNA microarray, which can be best done on fresh frozen tissue. In addition, method of real-time reverse transcriptase polymerase chain reaction (RT-PCR) can be used to assess the pre-selected specific number of genes or confirm expression of selected genes. The pre-select gene arrays determine about 21 predefined genes (included in multigene array) to predict response and recurrence to hormonal and drug therapies. On the other hand, the risk groups in gene pattern array can be classified more by using DNA microarrays into different groups according to gene expression: luminal A, luminal B, normal-like (mainly ER positive), basal-like (mostly ER negative), and HER2 positive (mostly ER negative). These subtypes showed different prognosis and response to treatment; however, basal-like, luminal B, and HER2-positive group showed worse outcomes. In addition, a good signature of 70 genes is related with low risk of metastasis while a poor signature of 70 genes is related with high risk of metastasis [5, 11, 88]. Human epidermal growth factor receptor 2 (HER2) is a member of epidermal growth factor receptor EGFR family. Overexpression of HER2 was found in 18–25% of breast cancer cases. In most cases, overexpression of HER2 is associated with high risk of nodal involvement, hormone receptor negativity, metastasis, and poor survival. Despite some uncertainties, HER2 status could be monitored in every patient scheduled to undergo hormonal/endocrine treatment [5, 11, 89]. Topisomerase II-alpha (topo IIα) is located adjacent to the HER2 oncogene at chromosome 17q12-q21, therefore it can predict HER-2-positive breast cancer, lymph node metastasis, and advanced stage of cancer. In addition, the status of topo IIα gene in the primary breast cancer is correlated with its status in the metastases [5, 90].

## Models of breast cancer

**Fig. 3:**
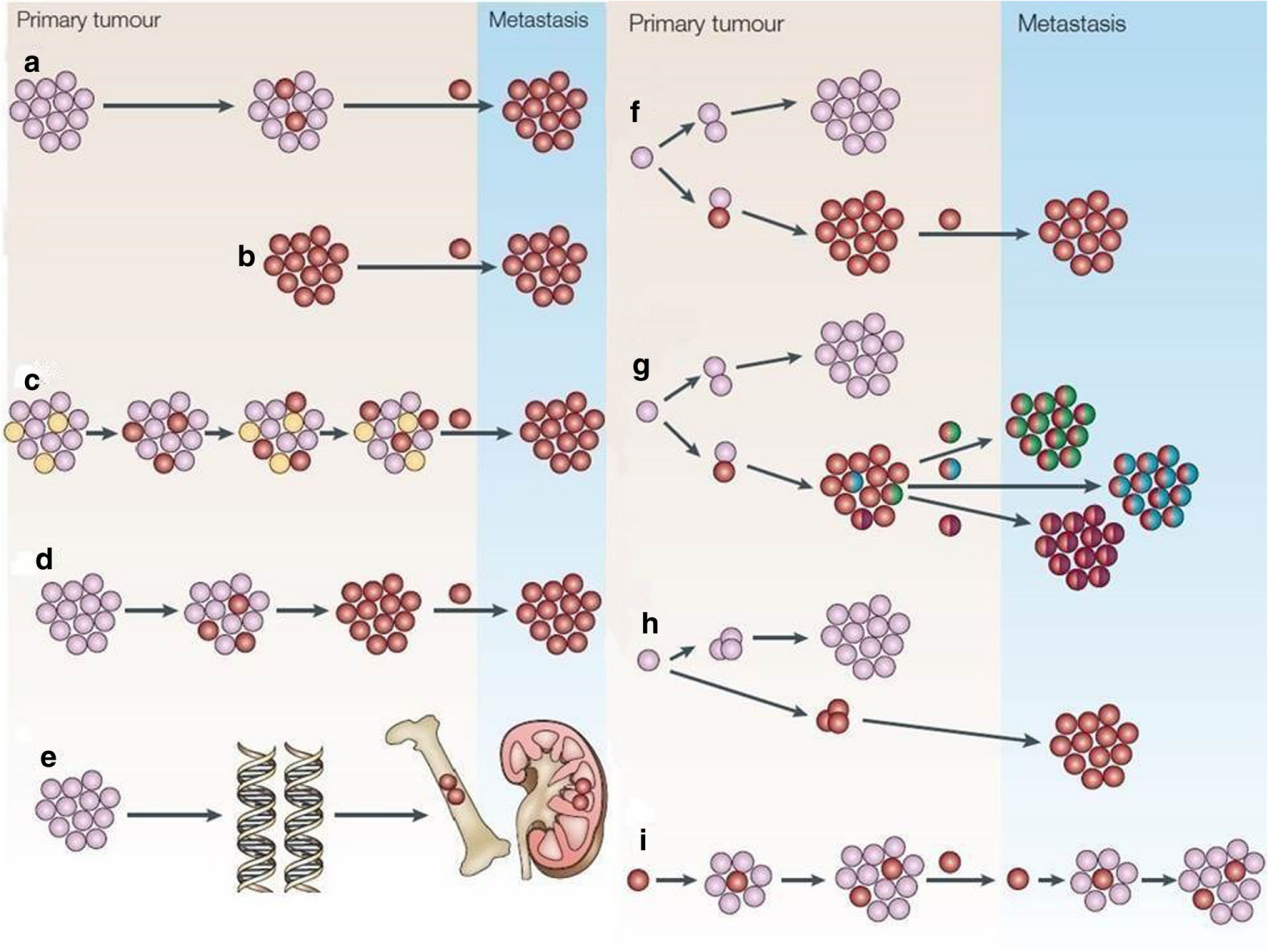
Models of MBC. **a** Tradition model of MBC. **b** Spontaneous metastasis assays. **c** Dynamic heterogeneity model. **d** Clonal dominance model. **e** Genometastasis hypothesis. **f** Gene expression profile. **g** Models of metastasis to lung, bone, and liver. **h** Parallel evolution model. **i** Breast cancer stem cells model. Pink represents non-metastasis breast tumor cells (good prognosis), red represents metastasis tumor cells (poor prognosis), yellow represents variant of tumor cells, green represents metastasis to bone, blue represents metastasis to liver, and purple represents metastasis to lung. Modified from [11]

Development of models of breast cancer was extremely important for the progress in discovering of new treatment approaches. The metastatic nature of tumor cells was discovered during the period 1970s–1980s by methods of “experimental metastasis” assays. It was reported that cells derived from outgrowths of metastatic cells have a higher metastatic activity than cells derived from the original cell line according to study of injecting intravenous metastatic cultured B16 melanoma cells into mice. Figure 3 illustrates three different models of MBC that have been developed [11, 28].

The first model of MBC cascade suggests that MBC occurs as either most cells of primary tumor have a low metastatic activity but acquire metastatic activity through additional somatic mutations during later stages of tumorigenesis, or spontaneous metastasis [11, 91–94]. The second model, which is a genetic expression analysis of breast cancer, suggested that MBC can occur due to the ability of cancer cells to acquire metastasis during the early stages of tumorigenesis; or more tissue-specific expression profile predicting the site of metastasis as lung, bone, and liver; or metastatic cancer cells can occur separately from the primary cancer cells during the early stages of oncogenesis (parallel evolution model); or only breast cancer stem cells have the ability to metastasize to distant areas of the body [3, 11, 92, 95–98]. The third model of MBC, which is the integrative model, predicted that the accumulation of somatic mutation and factors of tumor microenvironment such as fibroblasts, ECM, inflammatory cells, and blood vasculature can be responsible for metastasis of cancer. Furthermore, the breast cancer stem cells would induce the formation of new blood vessels at the site of metastasis and also induce a stromal response similar to that of their primary breast cancer. This model is based on studies of the fibroblast serum-response signature and prognostic markers like uPA/PAI1 and gene expression profile [3, 11, 99, 100].

## Treatments of metastatic breast cancer

**Fig. 4:**
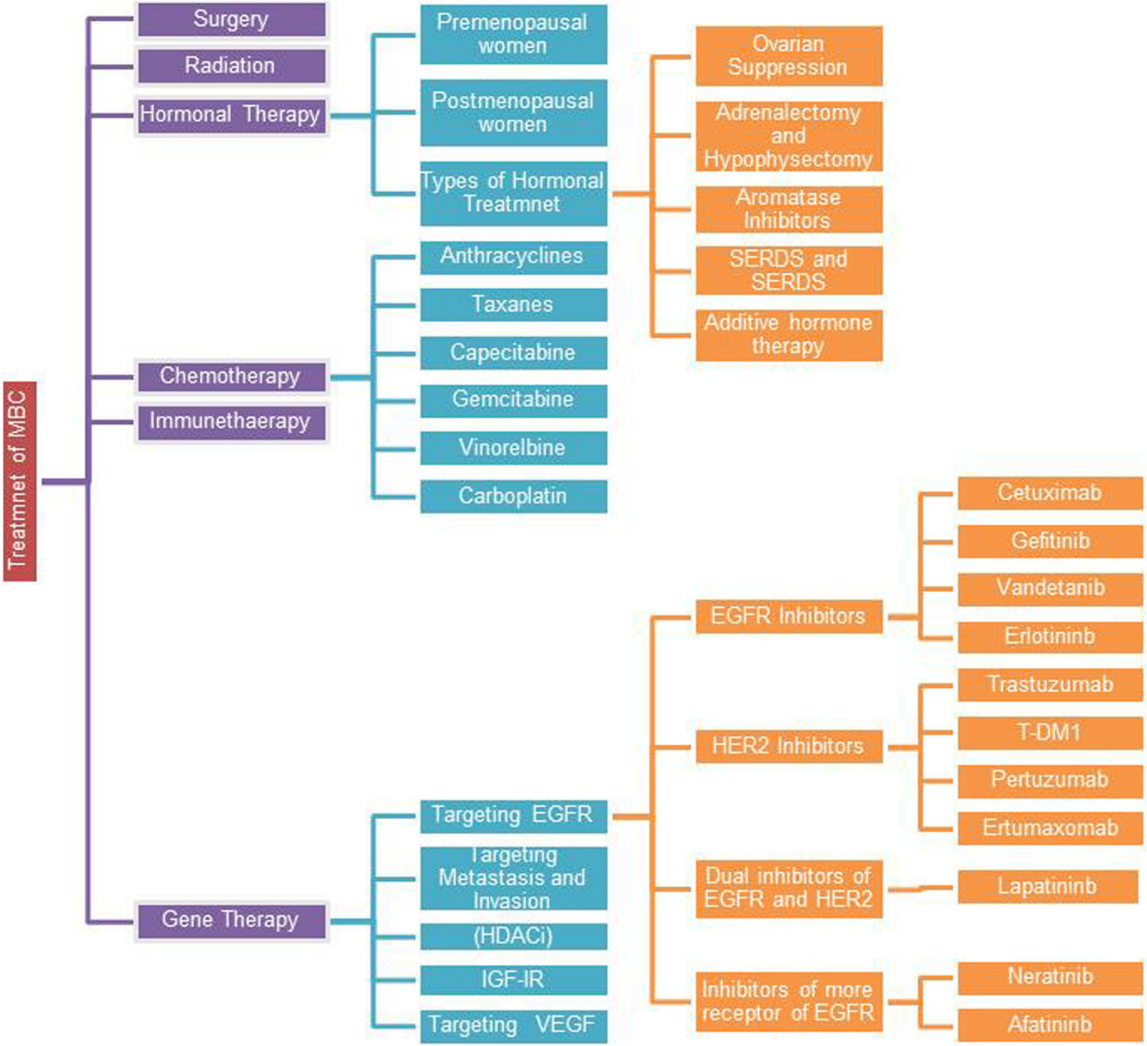
The most common types of MBC treatment

The goal of metastasis treatment is to prolong survival, palliate symptoms, and delay progression of the disease [5, 101]. Treatment of MBC varies with certain factors such as risk for toxicity, preferences of the patient, burden of the tumor, characterization of the tumor itself such as HER2 status and hormone receptor status, age, history of prior therapy, co-morbidities, degree of tumor-related symptoms, and metastasis sites. In fact, treatment of MBC can fall into three categories, such as surgery, chemotherapy, and hormonal therapy [102]. Combination of two or more regimens of MBC therapy can improve the quality of life and decrease the side effects associated with using single treatment. The most common types of treatment of MBC are summarized in Fig. 4.

### Surgery and radiation

Surgery can precede either hormonal therapy or chemotherapy or follow induction therapy. It is one of the common treatments of MBC disease especially in nodal dissection for locoregional and sentinel lymph node cases. The use of surgery can vary according to the clinical situation and characteristics of the patient; therefore, it can be used as a single treatment or in combination with chemotherapy or hormonal therapy to enhance the efficiency of MBC treatment [103]. In addition, surgery can improve the overall survival and reduce breast cancer mortality by preventing the potentially disabling complications (medullary compression, pathologic fractures), resecting of metastases (lung, ovary, liver), providing a symptomatic treatment (infiltration of the chest wall, local recurrence, bone pain), and excluding of another tumor or non-tumor diseases [103, 104]. On the other hand, surgery can cause an increase in the peripheral oxidative damage to macromolecules in the early postoperative period; therefore, perioperative antioxidant supplementation should be considered [105].

Radiation therapy is used in breast cancer following the mastectomy or surgery. However, radiotherapy showed relapse of about 7–12.6% among patients with 5 years and high resistance can occur; therefore, it is preferred to use a combination of radiotherapy and hormonal treatment especially if the size of the tumor is greater than 1 cm [4, 106, 107].

### Hormonal therapy

Hormonal or endocrinal therapy is an effective and a well-tolerated anti-cancer treatment. It is a systemic therapy and can be considered as the standard treatment in estrogen receptor-positive tumors of the early and late stage of breast cancer [108]. Hormonal treatment is also used in order to minimize the toxicity associated with other treatments. In addition, it can be given pre-operatively (neoadjuvant) or post-operatively (adjuvant), or during the MBC disease setting (palliative treatment) [109, 110]. However, sensitivity to hormonal treatment or resistance can occur among patients as side effects of this treatment [111].

#### Ovarian suppression

It is the first systemic therapy for any type of cancer and the oldest endocrinal therapy for hormone receptor-positive breast cancer that is recently been replaced by ovarian irradiation. Ovarian suppression is made by medical oophorectomy with the so-called luteinizing hormone-releasing hormone (LHRH) analogues or agonists such as goserlin, leuprolide, buserelin, and triptorelin [5, 112]. Although, certain LHRH receptors have been identified in breast cancer, LHRH agonists alone did not diminish the recurrence or mortality. Although the uses of ovarian suppression treatment is still controversial, this treatment is still required in patients with MBC and receiving LHRH agonist treatment and candidate for subsequently radiological or surgical ablation, as well as many subsequent second-line therapy options involving aromatase inhibitors that is needed for suppression of ovarian function [113].

#### Adrenalectomy and hypophysectomy

Adrenalectomy and hypophysectomy surgery are considered the first-line treatment in cases of postmenopausal women since the adrenal gland is a source of steroid production in postmenopausal women. Both these treatments are used in the management of MBC but with limited effect on morbidity and mortality. Therefore, an advance stage of medical adrenalectomy is introduced. Glucocorticoids treatment (prednisone/prednisolone 5-10 mg) daily showed a low toxicity and response when used in the MBC treatment with moderate doses. Moreover, a major discovery had been made with the introduction of amino glutethimide, which is an adrenal blocker as treatment of MBC. Furthermore, aminoglutethimide, which is unsuccessful antiepileptic drug, shows great antitumor effects due to its ability to inhibit aromatase enzymes [114].

#### Aromatase inhibitors

Aromatase inhibitors can inhibit aromatase enzymes that are responsible for the synthesis of estrogens from androgenic substrates that are produced by the adrenal glands and therefore used in the MBC treatment. These drugs are divided into two types: steroidal inhibitors (type 1 inhibitors) like exemestane and non-steroidal inhibitors (type 2 inhibitors) like anastrozole. Steroidal inhibitors are irreversible inhibitors of aromatase and analogues of adione while non-steroidal inhibitors bind reversibly to the haem group of aromatase. Although aminoglutethimide (the first-generation aromatase inhibitor) can suppress the estrogen and inhibit only aromatase enzyme, therefore the levels of circulating androgen were found to be not affected due to suppression of estrogens. In addition, because of the side effects and inconvenience of parenteral administration of the first generation, second, and third generation of the aromatase inhibitors such as anastrozole, formestane and letrozole were developed [115]. Moreover, the third generation of aromatase inhibitors showed a greater response than tamoxifen treatment alone [116, 117].

#### Selective estrogen receptor modifier and selective estrogen receptor downregulators

Tamoxifen is the most known drug of selective estrogen receptor modifier (SERMS) due to is antitumor activity and low toxicity. This drug is used as first-line treatment in premenopausal as well as postmenopausal women with MBC [112, 118, 119]. The regular dose of tamoxifen is 5 mg daily. Tamoxifen can interact with follicular maturation in premenopausal women leading to increase the plasma levels of estradiol about two- to threefold. Droloxifene and Toremifene with high dose are other drugs of SERMS group. They showed lower antitumor activity in premenopausal women but similar antitumor activity in postmenopausal women with tamoxifen [120]. Selective estrogen receptor downregulators (SERDS) are a novel group of drugs and fulvestrant is an example of this category of drugs. Fulvestrant is different from other SERMS drug in lacking any estrogen agonist activity and having a unique chemical structure. In addition, fulvestrant works by two mechanisms, namely downregulation of the receptor or blocking of the receptor. Moreover, fulvestrant with a dose of 500 mg has a great antitumor activity similar to tamoxifen; however, it is required to be administered parenterally [121].

#### Additive hormone therapy

Different treatments at high doses such as estrogens, androgens, and progestins can be used in MBC. Androgens were used in the treatment of breast cancer before nowadays treatments because most breast cancer receptors express androgen receptors at a level greater than 10 fmol/mg. However, androgen treatment shows a low response rate and is also associated with side effects such as hirsutism [122, 123]. Considering estrogen, it is used with higher doses (diethylstilbestrol 15 mg daily) in premenopausal and postmenopausal women with breast cancer. Estrogen can work as antitumor drug due to its high concentration that is greater than the optimal concentration for cell growth and showed similar antitumor activity similar to tamoxifen [124]. Although progestin can suppress the estrogens therefore used as antitumor treatment but is associated with weight gain as a side effect of its treatment. Both megestrol acetate with a dose of 160 mg daily and medroxyprogestrone acetate with a dose of 1000 mg daily showed similar antitumor activity similar to tamoxifen and aminoglutethimide [125].

### Chemotherapy

The uses of chemotherapy treatment vary according to different cases of MBC. Chemotherapy is considered as the first choice of MBC treatment in women who rapidly develop progressive visceral metastasis chemotherapy and having symptomatic or having hormone receptor-negative disease or having cancer resistant to endocrine therapy. In addition, chemotherapy is used as adjuvant treatment in patients with MBC who had received a local treatment and were at high risk of relapse as it is more beneficial in node-positive patients than node-negative patients. However, systemic chemotherapy showed less impact with age, severe side effects (nausea and vomiting), poor response, and overall did not improve the survival benefits of patients. The cytotoxic drugs can be administrated systemically (orally or intravenously) to kill cancer cells [126, 127].

#### Anthracyclines, taxanes, and carboplatin

Anthracyclines are the most common antitumor antibiotics used in the management of MBC. Epirubicin and doxorubicin antibiotics are examples of anthracyclines. They can work by different mechanisms such as impairing replication of DNA and mitochondrial function, generating oxygen-free radicals, activating of apoptosis and matrix metalloproteinase, as well as immune reactions [5, 128]. About 30–40% of MBC patients with anthracycline treatment showed response of survival within 22 months. The regimens containing anthracycline are better than regimens containing no anthracycline in the time of progression; however, they are associated with greater toxicity and there was no improvement in OS. The most common combinations of anthracyclines are CAF/CEF (cyclophosphamide 5-fluororacil plus epirubicin or doxorubicin) or AC/EC (doxorubicin/epirubicin plus cyclophosphamide). In addition, Myocet (liposome encapsulated doxorubicin) 75 mg/m^2^ every 3 weeks has shown to be less cardiotoxic than the traditional doxorubicin in MBC [5]. The use of anthracycline is limited because it is associated with acute toxicity such as myelotoxicity, alopecia, nausea, and vomiting and also due to their dose-dependent and irreversible cardio toxicity (over 1000 mg/m^2^ in the case of epirubicin or 450 mg/m^2^ in the case of doxorubicin) [128–130].

Taxanes are microtubule inhibitors that inhibit tumor angiogenesis and are considered as the first-line treatment in patients who are resistant to anthracycline or cannot receive more anthracycline treatment. Docetaxel and paclitaxel are examples of taxanes, which showed high response rate in anthracycline-resistant MBC cases [131, 132]. Taxanes can be used as single agent or in combination with other treatments such as the combination of anthracycline with taxanes that improve the quality of life better than anthracycline or taxanes treatment alone [5, 133]. In addition, combination of taxanes plus biological drugs such as trastuzumab showed improvement in overall survival in patients with MBC [134]. Furthermore, combination of lapatinib with docetaxel and trastuzumab can be used as a first-line treatment of HER2-positive MBC [135]. However, dose limiting and neuropathy are common side effect of taxanes therapy, which can be managed by delays and reductions of the dose [131].

Carboplatin is an alkylating agent or platinum compound used in the management of MBC that failed to response to other treatments. Carboplatin treatment can produce 20–35% of objective response rate (ORR) [136]. The combination of carboplatin to docetaxel/paclitaxel showed higher efficacy than carboplatin or taxane treatment alone. This combination showed higher efficacy in treating breast cancer that metastasis to brain tumor [137, 138]. In addition, combination of carboplatin plus trastuzumab/paclitaxel treatment showed superior efficacy for patients with HER2-positive MBC than using trastuzumab/paclitaxel alone [139]. Moreover, the combination of carboplatin with gemcitabine showed an effective treatment option for pretreated MBC patients [136, 140].

#### Capecitabine, gemcitabine, and vinorelbine

Capecitabine treatment is used in patients with disease resistant to anthracycline or taxanes treatment [141]. It is used as oral prodrug to generate 5FU in tumor tissue through activation pathway of thymidine phosphorylase. The oral solution of capecitabine was prepared to be similar to continuous infusion of 5FU [142, 143]. Capecitabine therapy showed 15–26% response rate with a dose of 1250 mg/m^2^ twice daily for 14 days [5]. The most common adverse effects of capecitabine therapy are nausea, hand-foot syndrome, , and in very rare cases alopecia and Myelo-suppression [142]. Capecitabine has more toxic effects than gemcitabine and vinorelbine treatment, so it is not preferred to be used alone [144]. Therefore, the combination of cpecitabine with other chemotherapy drug is used to prolong the duration of treatment, improve the efficacy, decrease the side effects, and maintain the therapy for patients with MBC [142, 145, 146]. Cabazitaxel or docetaxel plus capecitabine combination can be used to improve survival in patients with MBC recurring after anthracycline treatment than docetaxel treatment alone [5, 147].

Gemcitabine is a deoxycytidine-analogue antimetabolite and a nucleotide analogue that inhibits the synthesis of DNA [5, 133, 148, 149]. This drug is well-tolerated in elderly patients. In addition, it is related with low incidence of alopecia, nausea, and vomiting and the most common dose-limiting toxicities are thrombocytopenia and neutropenia [5]. Great efficacy, pharmacodynamics, and limited toxicity of gemcitabine make it an ideal agent for polychemotherapy combinations, specifically with vinorelbine, , and platinum derivates [150]. Gemcitabine plus paclitaxel combination showed 68% in overall response when used as first-line treatment and as 48% when used as second-line treatment [5]. In addition, gemcitabine plus transarterial chemoembolization can be used in the treatment of liver metastasis of breast cancer [151]. Moreover, gemcitabine can be used with bisphosphonate in the treatment of bone metastases of breast cancer [152]. Furthermore, low dose of gemcitabine plus cisplatin combination weekly showed efficacy and safety in the treatment of brain metastasis of breast cancer [153] and treatment of strongly pretreated MBC patients resistant to taxanes and anthracyclines treatments [154, 155].

Vinorelbine is a semisynthetic and third generation of vinca alkaloid [156]. It is safe and can be used alone or in combination with other drugs in the treatment of MBC [157, 158]. The oral dosage form of vinorelbine can be used alternatively to intravenous form in MBC treatment [159, 160]. Vinorelbine treatment showed 35–50% response when used as first-line treatment of MBC; however, the main adverse effects are superficial phlebitis, peripheral neuropathy, neutropenia, myelosuppression, leukopenia, and gastrointestinal toxicities [161]. Vinorelbine plus epiribicin combination showed a higher response rate (RR) and PFS but not OS [5]. The combination of oral vinflunine plus capecitabine treatment showed safe response and good antitumor activity in HER2/Neu-negative MBC patients who had failed to anthracyclines and taxanes [156, 162–164]. Moreover, vinorelbine plus gemcitabine combination showed better PFS compared with vinorelbine treatment alone [165]. Furthermore, low dose of oral vinorelbine plus temozolomide combination showed safe and effective effects in the treatment of brain metastasis of breast cancer [166].

### Immune therapy

In most cancers, the immune microenvironment is a balance of immune cells between mediating and preventing the destruction of tissue. Type I immunity such as CD4^+^ T cells that secrete cytokines like TNF-α, IFN-γ, CD8^+^, and interleukin (IL)-2 cytotoxic T cells support the destruction of tissue environment. The IL-2 activation of T cells induces a regression of MBC in renal cancer and melanoma. In addition, the abundance of tumor-infiltrating leukocytes, CD3^+^, and CD8^+^ T lymphocytes have been related with PSF and OS of breast cancer patients. Three immune metagenes that represent the tumor-infiltrating populations and strongly associated with high survival of MBC patients are (1) B cells/plasma B cells determined by the high expression of IgG antibody isotype-related genes, (2) a monocyte/dendritic cell population determined by the expression of myeloid specific markers and a host of major histocompatibility complex class II antigen-presenting molecules, and (3) T cell/natural killer cell-specific population determined similarly. Furthermore, signal transducer and activator of transcription 3 (Stat3) controls genes that are involved in cell proliferation and in the production of angiogenic and antiapoptotic factors. Consequently, ablating Stat3 signaling in breast cancer cells may represent an effective approach in immunotherapy of breast cancer growth and metastasis that can result in induction of a cellular senescence program. However, such approach requires extensive immunotherapy research. On the other hand, type II immune system composed of CD4^+^ T cells that secrete cytokines like IL-4, IL-6, and IL-10 which in turn decrease the acute inflammatory response and prevent the proliferation of cytotoxic T cells. Moreover, CD4^+^ T cells showed a strong relationship with the progression of the tumor and tumor-specific CD8^+^ T cells. It was shown that mutation in cytotoxic T cell epitopes within the tumor antigen resulted in the progression of the tumor. An interesting multipronged approach to cancer treatment combines natural killer (NK) cell and cytotoxic T cells-based autologous immune enhancement therapy (AIET) with conventional approaches of treatments such as surgery, chemotherapy, and radiotherapy as well as other modalities like hyperthermia, proton beam therapy, and also low-dose chemotherapy. It seems that such complex approach can be effective in advanced cancers which are refractory to conventional simpler therapeutic approaches. Furthermore, treatment of breast cancer with biologic drugs can induce type I immunity microenvironment and improve the therapy or decrease the recurrence of breast cancer [167, 168].

### Gene therapy

Genes that control metastasis of the cancer is divided into two groups: metastasis suppressor genes (MSGs) and metastasis promoter genes (MPGs). The normal function of MSGs is preventing cells from divisions or proliferation and inhibiting the spread and growth of cancer while MPGs do the opposite. In addition, the concept of metastasis-related gene is known in 1970, but the search of MSGs started in the mid-1980. Since MBC is a cascade of signals, targeting these signals of genes can potentially help to improve MBC therapy [5].

#### Epidermal growth factor receptor and their inhibitors

##### Cetuximab, gefitinib, vandetanib, and erlotinib

The epidermal growth factor receptor (EGFR) is a transmembrane tyrosine kinase receptor which triggers the phosphatidylinositol 3-kinase (PI3K/Akt) pathway on activation. EGFR is also a member of the HER family that is membrane-bound receptor tyrosine kinases (RTKs) and composed of four structurally related receptors: EGFR, HER2, HER3/ErbB3, and HER4/ErbB4. EGFR has the ability to stimulate motility, proliferation of cells, angiogenesis, and metastasis of breast cancer. About 50–75% of breast cancer cells and about 45% of MBC patients have been shown to be EGFR positive resulting in more aggressive tumor than cells lacking this factor. Consequently, inhibitors of EGFR (antibodies or small molecules) can be used in the treatment of MBC [169–174].

Cetuximab is a chimeric anti-EGFR monoclonal antibody. HER1 receptor has a role in mediated cell signaling which is related to proliferation of the tumor, angiogenesis, metastasis, and apoptosis. In addition, overexpression of HER1 receptor and its ligand is noticed in multiple human malignancies such as lung cancer, pancreatic cancer, colorectal cancer, and breast cancer. Cetuximab has a synergistic effect with radiotherapy or chemotherapy and can be used in the treatment of triple-negative breast cancer cells (TNBCs) that are overexpressed EGFR. In addition, weekly combination of cetuximab with taxane can be used for patients with TNBCs [175–178].

Gefitinib is a small molecule drug that irreversibly inhibits EGFR receptor (tyrosine kinase inhibitor) [169, 170]. The major problem associated with gefitinib treatment is the development of resistance; therefore, combination of gefitinib with other drugs can be used to overcome this problem [170]. Gefitinib can be used in HER2 MBC patients in combination with trastuzumab and docetaxel to reduce the resistance and overcome toxicities associated gefitinib [179].

Vandetanib is an oral active antagonist of EGFR (ErbB1or HER1), vascular endothelial growth factor receptor-2 (VEGFR-2), and RET kinase. Vandetanib can be used in the treatment of thyroid cancer, prostate cancer, non-small cell lung cancer, breast cancer, and colorectal cancer. This drug received its first global approval for the treatment of metastatic medullary thyroid cancer in the USA on 6 April 2011. In MBC, vandetanib with docetaxel combination showed greater efficacy than placebo combined with docetaxel only. However vandetanib with 100 or 300 mg/day did not show a good response in the treatment of patients with previously treated MBC. Diarrhea, nausea, fatigue, abnormal hepatic function, and hyperglycemia are side effects associated with using vandetanib therapy in breast cancer [180, 181].

Erlotinib is an orally potent EGFR inhibitor. It is used for the treatment of pancreatic cancer and non-small cell lung cancer. However, it showed less activity in MBC women therapy. In addition, using erlotinibcan with bendamustine in metastatic triple-negative breast cancer produced prolonged and sever lymphopenia. Furthermore, combination of erlotinib with docetaxel/capecitabine can be used in MBC treatment [182, 183].

##### Inhibitors of multiple receptors of EGFR family: neratinib and afatinib

Neratinib is an irreversible pan-tyrosine kinase inhibitor that also demonstrates the activity against HER1, HER2, and HER4. Neratinib is a low molecular weight, orally administrated antitumor drug that is used in patients with advanced HER2-positive breast cancer which early have been exposed to trastuzumab or are resistant to EGFR inhibitors. Neratinib is about 12- to 16-fold more potent than lapatinib in inhibiting proliferation of HER2-positive breast cancer cells [184]. Combination of neratinib with vinorelbine showed a great antitumor activity in HER2-positive MBC patients [157]. The most common adverse effects associated with neratinib treatment alone are nausea, diarrhea, vomiting, and fatigue [184–187].

Afatinib is an oral, small molecule anilinoquinazoline compound which is a highly selective inhibitor of EGFR/HER1, HER2, and HER4 tyrosine kinase activity. This drug can be used alone or in combination with other treatment in HER2-positive breast cancer. Although afatinib demonstrates a limited effect in HER2-negative breast cancer patients, it can be combined with vinorelbine or trastuzumab in the treatment of HER2-positive MBC. Moreover, afatinib can be used with the standard neoadjuvant therapy that includes anthracycline/taxane and trastuzumab in the treatment of HER2-positive operable or locally advanced breast cancer. The adverse effects of afatinib are mainly associated with gastrointestinal toxicities [185, 188–191].

#### HER2 inhibitors: trastuzumab, ado-trastuzumab emtansine, pertuzumab, and ertumaxomab

Trastuzumab is a humanized monoclonal antibody directed against HER2 glycoprotein (anti-HER2/neu treatment). The HER2 is overexpressed in 20–25% of human breast cancers leading to increase the aggressiveness of the tumor and decrease OS. Trastuzumab showed about 35% of response in the treatment of MBC [100, 192–194]. In addition, trastuzumab recently have been used alone or in combination with chemotherapy in the treatment of MBC in patients that overexpress HER2 protein. Trastuzumab showed a good effect in women with HER2/neu-positive disease compared with women with HER2/neu-negative disease [185, 195–200]. In addition, trastuzumab plus paclitaxel combination showed higher RR and OS in MBC patients pretreated with an anthracycline [201]. Moreover, the combination of trastuzumab and docetaxel can be used for treating patients with HER2-positive or HER2-negative overexpressing metastatic breast cancer. This combination showed good results; however, with a little more toxicity, time to treatment failure, time to progression, rate and duration’s response, and overall survival [202, 203]. Furthermore, combination of trastuzumab with other cytotoxic agents such as anthracycline, carboplatin, taxanes, vinorelbine, and gemcitabine were effective when used as first- or second-line treatment especially in HER2-positive MBC patients [204–206].

Ado-trastuzumab emtansine is a conjugate of the antibody (trastuzumab) with the drug (emtansine, anti-microtubule agent). Trastuzumab is considered the backbone that attached to emtansine by stable linker to deliver chemotherapy agent to cancerous tissues that overexpressed HER2 without adverse side effects on normal cells. Ado-trastuzumab emtansine (T-DM1) has the ability to combine the cytotoxic effects of emtansine with the antitumor activity of trastuzumab (HER2 inhibitor). In addition, T-DM1 has been shown to improve PFS and OS in HER2-positive MBC. Moreover, T-DM1 can be used effectively in the treatment of HER2-positive MBC patients that previously received trastuzumab, taxane, and lapatinib. Cardiotoxicity, thrombocytopenia, and increased liver enzymes are the main adverse side effects associated with T-DM1 therapy [207–211].

Pertuzumab is a humanized monoclonal antibody that blocks the dimerization of HER receptors leading to decrease the intracellular signaling of HER2 receptor. Pertuzumab is different from trastuzumab in that it binds to a different domain of HER2. This drug can be used alone or in combination with trastuzumab and docetaxel in the treatment of HER2 MBC patients showing prolonged PFS and improved OS. Furthermore, pertuzumab showed acceptable tolerability and no evidence of increasing the risk of cardiotoxicity [212–217].

Ertumaxomab represents a monoclonal antibody targeting HER2/neu and CD3 on T cells. It is able to stimulate the recognition and destruction of cancer cells by different immunologic mechanisms such as dendritic cells (DC), dendritic cell cytokine 1 (DC-CK1), leukocyte function associated antigen (LFA), antibody-dependent cellular cytotoxicity (ADCC), and tumor necrosis factor-α (TNF-α) and cluster of differentiation (CD). Ertumaxomab in the treatment of breast cancer showed a strong immunologic response; however, the most common adverse effects of ertumaxomab are vomiting, fever, elevated liver enzymes, and lymphocytopenia [185, 218–220].

#### Dual inhibitors of EGFR and HER2: lapatinib

Lapatinib is an oral inhibitor for both HER2 and EGFR1. It can be used alone or in combination with other pharmaceuticals in the treatment of HER2-positive MBC [171, 185, 221]. The combination of lapatinib with carboplatin represents an effective therapy for brain metastasis of HER2-positive breast cancer and especially in cases when trastuzumab has no effect [222]. The combination of lapatinib with capecitabine is more effective in patients who received less than two regimens for MBC and are naive to capecitabine [223–226], also the oral combination of these therapies can be used in HER2-positive metastatic brain cancer form [227, 228]. Moreover, the combination of lapatinib plus vinorelbine showed moderate efficacy among MBC patients with overexpression of HER2 [229]. Furthermore, the combination of lapatinib plus trastuzumab showed higher efficacy especially in metastasis brain cancer when compared with a single treatment alone [194, 230].

#### Inhibition of the urokinase-type plasminogen activator system

The urokinase-type plasminogen activator (uPA) and its receptor uPAR play an important role in the angiogenesis, invasion, and metastasis of the tumor. uPA is a member of the serine protease family which catalyzes the conversion of inactive zymogen plasminogen to its active form plasmin. When uPAR stimulate direct plasmin-mediated proteolysis, the plasmin degrades most components of the ECM like fibronectin, laminin, and collagen that are produced by tumor surrounding stroma and tumor cells. Binding of uPA to its receptor stimulates activation of other proteinases like metalloproteases (MMPs). Moreover, uPA is associated with chemotaxis, cell proliferation, and angiogenesis elevation in malignant tumor. Therefore, inhibition of uPA and its receptor uPAR represents an attractive approach for MBC treatment. The drug candidate WX-UK1 is a 3-amidinophenylalanine-based inhibitor of the uPA system that is used to inhibit the metastasis capacity of tumor cells in vitro. Combination of WX-UK1 with capecitabine can also be used in MBC treatment [76, 231].

#### Matrix metalloproteinases inhibitors

The MMPs, especially MMP-2 and MMP-9, have been involved in several types of cancer and their metastasis such as ovarian, colorectal, and breast cancers. MMPS are able to modulate the progression of the tumor in managing the epithelial-mesenchymal transition, invasion, metastasis, and growth of the tumor; participate in pre-metastatic niche formation; and inducing an inflammatory response. Also, MMPs can have a dual role during formation of the blood vessels and apoptosis evasion. High MMPs content in the model of human osteosarcoma cell destroy ECM; therefore, the level of MMPs is related with metastasis of the tumor. In addition, MMPs stimulate the migration of endothelial cells and facilitate the formation of new blood vessels. Moreover, MMPs showed strong correlation with uPA and negative correlation between uPA/MMPs with inhibitors of metalloproteinases (TIMPs). BAY 12-9566 is an inhibitor of MMP-2, MMP-9, and MMP-3 that showed no musculoskeletal effects and well tolerated in patients with solid cancer. In addition, combination of BAY 12-9566 with etoposide, doxorubicin, carboplatin, 5-fluorouracil, and leucovorin can be used in cancer therapy. Moreover, other MMP inhibitors, such as asmarimastat, solimastat, metastat, prinomastat, BMS 275291, and neovastat, are currently under the clinical trials [232–235]. Figure 8 shows the role of MMPs in carcinogenesis.

#### Histone deacetylase insulin-like growth factor and insulin-like growth factor inhibitors

The Histone acetyl transferases (HATs) and histone deacetylases (HDACs) play an important role in maintaining the balance between the acetylated and deacetylated states of histones, gene expression, and modification of chromatin structure. In addition, inactivation of HATs is related with tumorigenesis. The histone deacetylase inhibitors (HDACi) are new class of anticancer agents that stimulate differentiation/apoptosis and inhibit the proliferation of cancer cells by inhibiting the function of HDACs. HDACi sensitizes tumor cells to topoisomerase inhibitors by increasing their access and binding to DNA. In addition, HDACi have been related with a transcriptional down regulation of ER in ER positive tumor cells. The combination of HDACi vorinostat with doxorubicin showed a significant antitumor activity in prostate, melanoma, and breast cancer. Furthermore, combination of another HDACi–valproic acid, with epirubicin improved their antitumor activity in patients pre-treated with anthracyclines [237–239].

The insulin-like growth factor inhibitor (IGF-IR) plays a major role in the proliferation and metastasis of different types of cancer like pancreatic, colon, prostate, and breast cancer. IGF-IR consists of an intracellular β subunit responsible for signal transduction and an extracellular α ligand-binding subunit and binds to IGF-1 and IGF-2 ligand-activated IGF-IR. High levels of IGF-I are strongly related with high risk of breast cancer. The overexpression of IGF-I leads to improved survival, proliferation signals for the breast tumor, and develop resistance to cancer treatment. In contrast to normal tissues, IGF-IR is overexpressed in about 50% of primary breast cancer tissues. Therefore, inactivation of IGF-IR results in decreased growth and metastasis of breast tumor in vivo. IMC-A12 is human monoclonal antibodies that bind with high affinity to IGF-IR and prevent the activation of ligand-dependent receptor and downstream signaling. BMS-554417 is novel IGF-IR that has a pronounced proapoptotic and antiproliferative activity in vitro and in vivo. In addition, IGF-IR can be used in the treatment of breast cancer in combination with cytotoxic drugs (e.g., aromatase drugs) or hormonal treatment. Furthermore, IGF-IR can be used in combination with EGRF inhibitors like leptin, lapatinib, and erlotinib to improve treatment of MBC [240–242].

#### Vascular endothelial growth factor

The vascular endothelial growth factor (VEGF) is a potent inducer of cell invasion, migration, vascular permeability, and vessel formation. There are five glycoproteins VEGFA, VEGFB, VEGFD, and placental growth factor that act by three receptor tyrosine kinases VEGFR-1, VEGFR-2, and VEGFR-3. Consequently, drugs targeting VEGF can potentially be used for treatment of different cancers including the MBC.

### Drug and gene delivery for treatment of breast cancer metastasis

Metastasis represents a growth of secondary malignancies in a distance from the primary tumor site. Several mechanisms may be responsible (often in combinations) for the movement of cancer cells from an original location and establishing remote colonies [243]. Such growth may be achieved by direct invasion of cancer cells into neighboring tissues, permeation via lymphatic vessels into lymph nodes, embolism through blood vessels, etc. Based on the origin and mechanisms of metastases, different methods of treatment of metastases in general and breast cancer metastases in particular can be grouped into two distinct clusters. First, since metastatic cells are originated from the primary tumor and therefore consist of the same type (or mixture of several subtypes) of cancer cells as the main formation, the same treatment methods and pharmacological agents potentially can be used for therapy both primary and metastatic cancers. Theoretically, all described above types of treatment approaches (hormonal, immune, gene therapy, or/and chemotherapy) can be used for treating both primary and metastatic cancers. However, in order to effectively kill spread metastatic cells, drugs or/and other active substances (antibodies, nucleic acids, peptides, etc.) in most cases should be delivered systemically and therefore they potentially can induce severe adverse side effects upon healthy tissues. Consequently, an effective and relatively safe treatment of metastases should provide a targeted delivery of active component(s) specifically to circulating cancer cells or/and induce cell death only in cancer cells protecting healthy organs, tissues, and cells. It is also important to deliver these agents to organs with metastases (e.g., brain via poorly penetrated blood-brain barrier or locally) on late stages of cancer. Local/topical treatments, surgery, or radiation may also be used to prevent or treat symptoms of MBC. For a more detailed discussion of different mechanisms of targeting of delivery systems to cancer, the reader is referred to our published reviews [244–247]. Second, in order to keep cancer cells within the limits of the primary tumor and prevent their spreading via the circulation/lymphatic drainage, one can inhibit the above described mechanisms of metastases. The most promising attempts to block the release of cancer cells from the primary solid tumor and their accumulation in organs are based on several approaches including (1) neutralizing of chemokines and their interactions with corresponding receptors [248], (2) blocking of angiogenesis (also used for treatment of primary tumors) [249–253], (3) axillary treatment [254] and lymphadenectomy [255], (4) targeted therapies and radionuclides [256], (5) gene and drug therapy of epithelial-to-mesenchymal transition (EMT) and mesenchymal-to-epithelial transition (MET) [257], and (6) corticosteroids, anti-epileptic drugs, and radiotherapy [258], and few others. In summary, the second approach—inhibiting mechanisms of metastasis—is currently only in an initial phase of the development. It should be stressed that combinatorial complex approaches potentially can demonstrate a high potential in treatment of primary and metastatic breast cancers [247, 259–262].

#### Combinatorial delivery by liposomes of gefitinib and small interfering RNA targeted to EGF receptors for treatment of TNBC

**Fig. 5:**
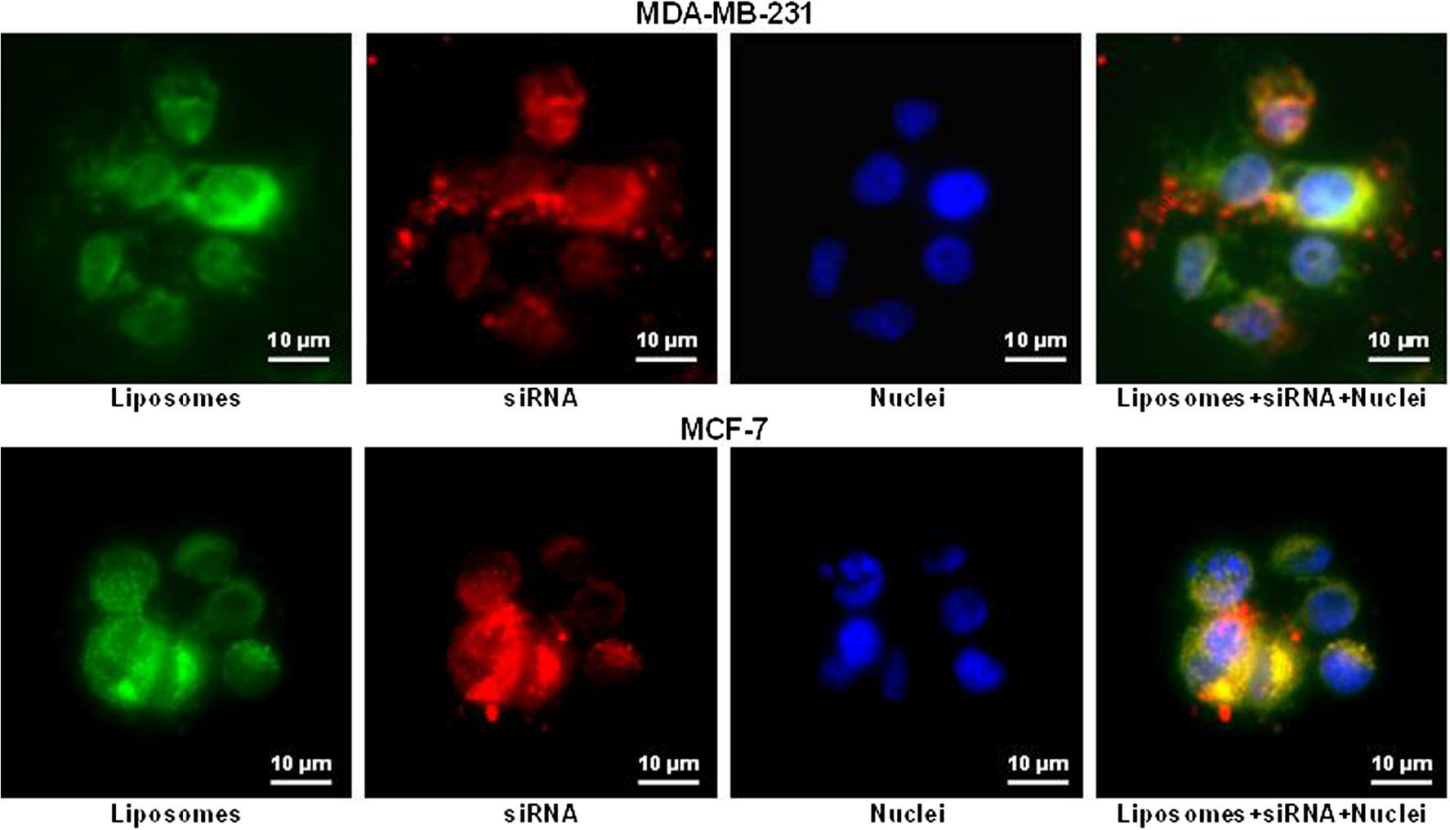
The cellular internalization of siRNA delivered by liposomes**.** Representative images of human breast cancer (MDA-MB-231 and MCF-7) cells incubated within 24 h with liposomes (green fluorescence) containing siRNA (red fluorescence). Cell nuclei were stained with nuclear-specific dye (DAPI, blue fluorescence). Superimposition of red and green colors gives yellow color

**Fig. 6:**
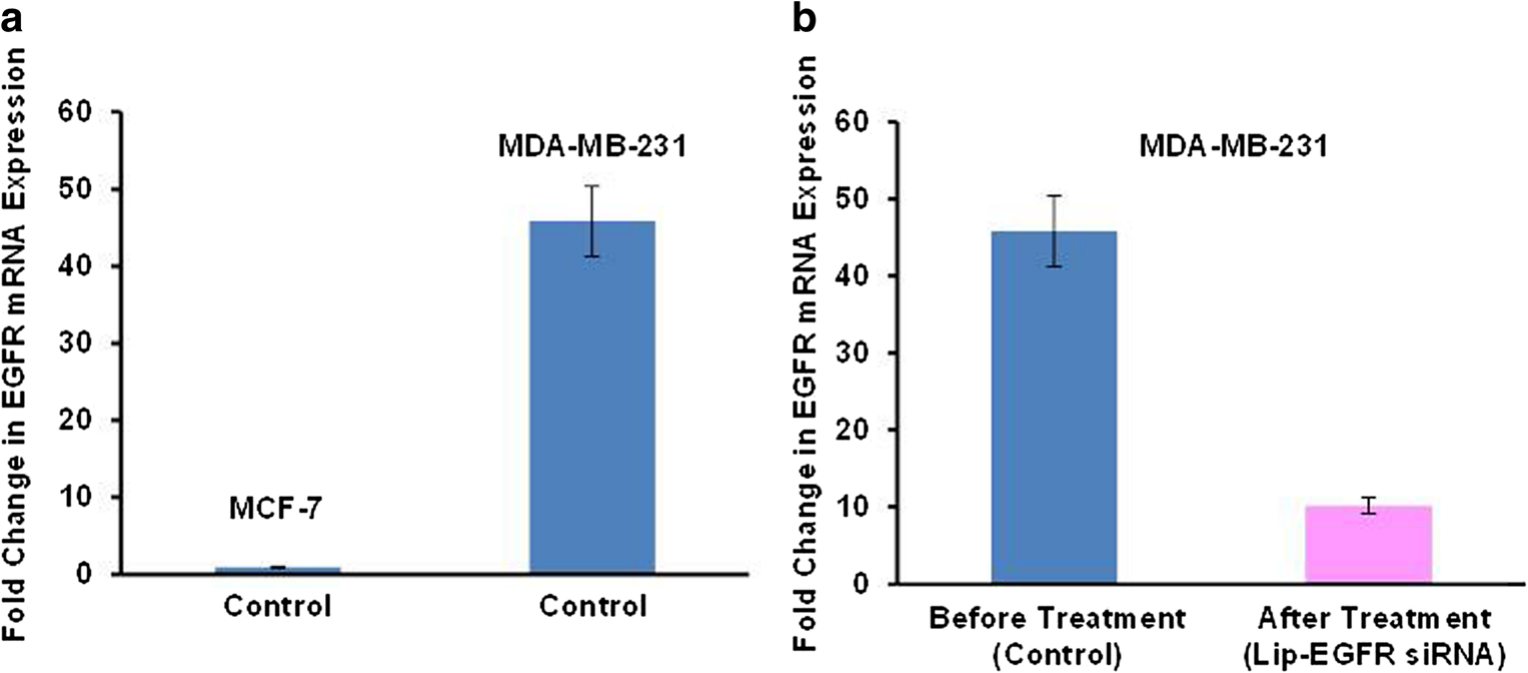
The expression of EGFR mRNA. The relative quantity of EGFR gene expression in MCF-7 and MDA-MB-231 human breast cancer cells was calculated by the 2^(DDCt)^ method using quantitative PCR. The levels of gene expression were represented as a fold change. Means ± SD are shown. **a** Expression of EGFR in MCF-7 and MDA-MB-231 cells incubated with media (control). **b** MDA-MB-231 cells before and after treatment. Cells were incubated within 24 h with liposomal siRNA targeted to EGFR mRNA (Lip-EGFR siRNA)

Small interfering RNA (siRNA) represents an attractive tool for inhibition of a specific mRNA and corresponding protein. In our lab, we used siRNA targeted to EGFR receptors in combination with gefitinib for the effective treatment of TNBCs. Both the siRNA and gefitinib were delivered by cationic and neutral liposomes, respectively. The toxicity of this combination for sensitive MCF-7 and triple-negative MDA-MB-231 human breast cancer cells was studied with appropriate controls (Figs. 5 and 6).

**Fig. 7:**
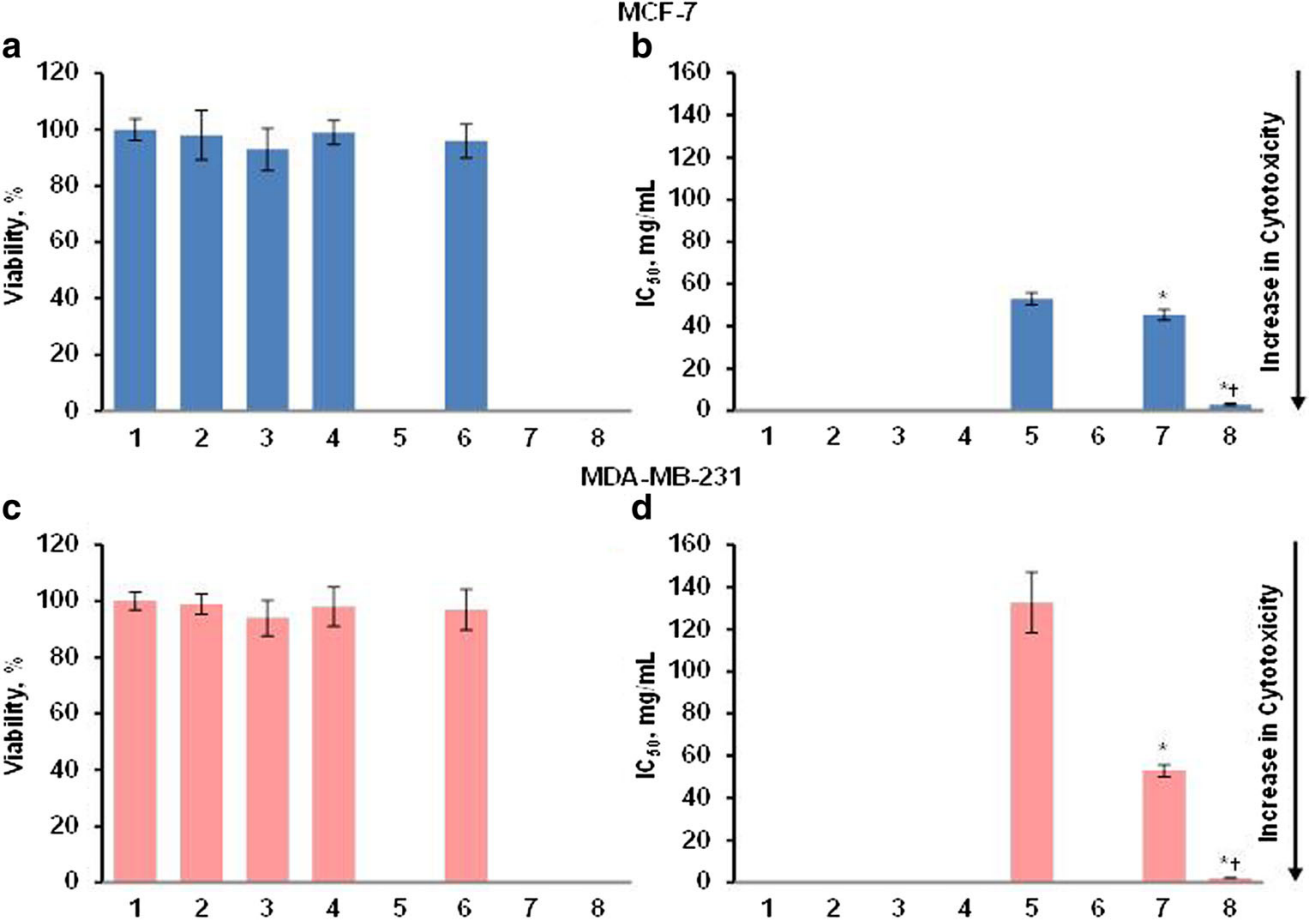
Viability of MCF-7 and MDA-MB-231 human breast cancer cells incubated 24 h with the indicated formulations. **a**, **c** Cytotoxicity of formulations that do not contain gefitinib in MCF-7 (**a**) and MDA-MB-231 (**c**) cells. **b**, **d** Cytotoxicity of formulations that contain gefitinib in MCF-7 (**b**) and MDA-MB-231 (**d**) cells. (1) Control (fresh media); (2) liposomes neutral; (3) liposomes cationic; (4) naked siRNA targeted to EGFR mRNA; (5) free gefitinib; (6) liposomal siRNA targeted to EGFR mRNA; (7) liposomal gefitinib; (8) liposomal siRNA targeted to EGFR mRNA + liposomal gefitinib. Means ± SD are shown. *P < 0.05 when compared with free gefitinib; ^†^*P* < 0.05 when compared with liposomal gefitinib

**Fig. 8:**
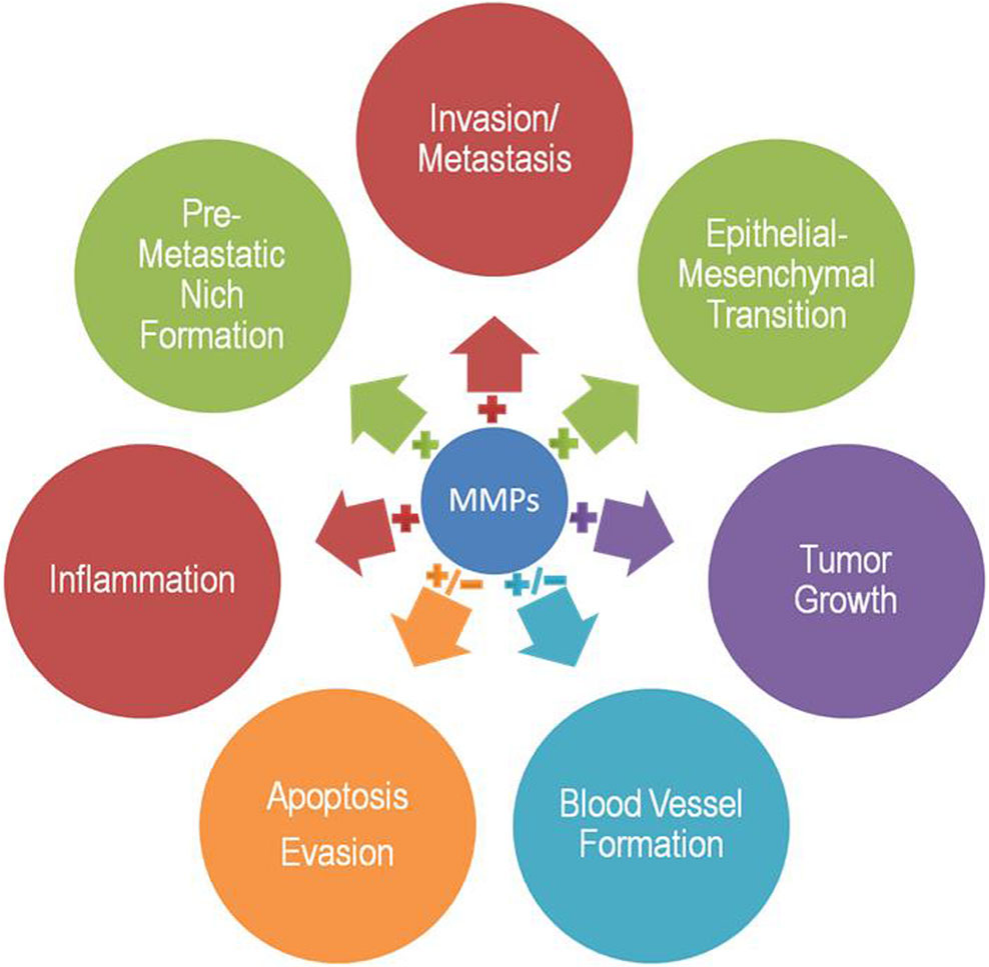
The role of MMPs in the progression and metastasis of cancer. Modified from [236]

It was found that empty liposomes, naked, and liposomal siRNA were not toxic for both cell types. Free gefitinib was significantly less potent in triple-negative breast cancer cells (compare bar 5 in Fig. 7b, d). The delivery of the drug by liposomes significantly enhanced its toxicity in both cell types. It was also found that the mixture of liposomal gefitinib with liposomal siRNA targeted to EGFR were significantly more toxic in both cell lines when compared with liposomal gefitinib alone. Consequently, the combination of EGFR siRNA with other EGFR small molecule inhibitor(s) delivered by liposomes represents a potent attractive approach for treatment of triple-negative breast cancer (Fig. 8).

## Conclusion

Breast cancer is divided into several groups according to IHC: ER positive and EGFR negative, HER2 positive which is either ER negative or ER positive and triple negative that is ER, PR, and HER2 negative. MBC is the advance stage of breast cancer and is associated with increase the mortality. Cancer prognostic factors are biological molecules that are produced either by the cancer cells or by human tissues in response to cancer. The biomarkers of cancer can detect the cells of cancer either by secretions like stools, sputum, urine, or nipple discharge or in the circulation like plasma, whole blood, or serum or in other human biological like ductal lavage and breast cyst fluid breast in the case of breast cancer. In addition, different factors like angiogenesis and cathepsin D can be used in the prognostic, predictive, and pharmacodynamics of breast cancer. Treatment of MBC still represents a challenge and involves different approaches including surgery, hormonal treatment, chemotherapy, and immunotherapy. A special treatment is required for TNBC. We proposed an innovative approach for the treatment of this type of breast cancer. It includes a combination of siRNA targeted to EGFR mRNA delivered by liposomes with liposomal gefitinib. It was shown that siRNA effectively suppressed resistance of TNBCs to gefitinib and, consequently, enhanced the efficacy of the treatment demonstrating a high potential of liposomal EGFR siRNA in combination with liposomal gefitinib for treatment of triple-negative breast cancer.

## Abbreviations

ADCC: Antibody-dependent cellular cytotoxicity
AIET: Autologous immune enhancement therapy
ALND: Axillary lymph node dissection
BMDCs: Bone marrow-derived dendritic cells
CD: Cluster of differentiation
CTCs: Circulating tumor cells
DC: Dendritic cells
DC-CK1: Dendritic cell cytokine 1
ECM: Extracellular matrix
EGFR: Epidermal growth factor receptor
ER: Estrogen receptor
HATs: Histone acetyl transferases
HDACi: Histone deacetylase inhibitors
HDACs: Histone deacetylases
HER2: Human epidermal growth factor receptor 2
IGF-IR: Insulin-like growth factor inhibitors
IHC: Immunohistochemical
IL: Interleukin
LFA: Leukocyte function-associated antigen
LHRH: Luteinizing hormone-releasing hormone agonist
LVI: Lymphovascular invasion
MAI: Mitotic activity index
MBC: Metastatic breast cancer
MFI: Metastatic-free interval
MMPs: Matrix metalloproteinases
MRI: Magnetic resonance imaging
NK: Natural killer cells
ORR: Objective response rate
OS: Overall survival
PAI1: Plasminogen activator inhibitor type 1
PCNA: Proliferating cell nuclear antigen
PFS: Progression-free survival
PR: Progesterone receptor
RR: Response rate
RT-PCR: Real-time reverse transcriptase polymerase chain reaction
SERDS: Selective estrogen receptor downregulators
SERMS: Selective estrogen receptor modifier
SLNB: Sentinel lymph node biopsy
SPF: S-phase fraction
Stat3: Signal transducer and activator of transcription 3
T-DM1: Ado-trastuzumab emtansine
TIMPs: Inhibitors of metalloproteinases
TLI: Thymidine labeling index
TNBC: Triple-negative breast cancer
TNBCs: Triple-negative breast cancer cells
TNF-α: Tumor necrosis factor-α
topo IIα: Topisometase II-alpha
uPAR: Urokinase-type plasminogen receptor
uPI: Urokinase-type plasminogen activator
VEGF: Vascular endothelial growth factor family
VEGF-2: Vascular endothelial growth factor receptor-2
